# State-church partnerships as an innovative strategy in healthcare delivery for universal health coverage in sub-Saharan Africa: a scoping review

**DOI:** 10.1093/heapol/czaf082

**Published:** 2025-10-29

**Authors:** Joseph Atta Amankwah, Emmanuel Kwasi Afriyie, Munawar Harun Koray, Kofi Mensah Akohene, Peter Agyei-Baffour

**Affiliations:** School of Health Management and Planning, School of Public Health, College of Health Sciences, Post Office Box LG 112, KNUST, Kumasi, Ashanti Region, Ghana; Methodist Faith Healing Hospital, Post Office Box 1256, Ankaase, Ashanti Region, Ghana; Komfo Anokye Teaching Hospital, Post Office Box 1934, Kumasi, Ashanti Region, Ghana; Southern Medical University, No. 1023-1063 South Shatai Road, Baiyun District, Guangzhou City, Guangdong Province 51051, China; Southern Medical University, No. 1023-1063 South Shatai Road, Baiyun District, Guangzhou City, Guangdong Province 51051, China; Upper West Regional Health Directorate, Post Office Box 132, Wa, Upper West Region, Ghana; School of Health Management and Planning, School of Public Health, College of Health Sciences, Post Office Box LG 112, KNUST, Kumasi, Ashanti Region, Ghana; School of Health Management and Planning, School of Public Health, College of Health Sciences, Post Office Box LG 112, KNUST, Kumasi, Ashanti Region, Ghana

**Keywords:** state-church partnerships, faith-based organizations, healthcare delivery, universal health coverage, sub-Saharan Africa

## Abstract

Universal Health Coverage (UHC) remains a critical goal in sub-Saharan Africa (SSA), where healthcare systems face significant challenges. State-Church Partnership have emerged as an innovative strategy to address gaps in healthcare delivery, leveraging the extensive networks of Faith-Based Organizations to provide essential services, particularly in remote areas.This scoping review aimed to examine the existing models of State-Church Partnerships in healthcare delivery within SSA, their impact on UHC advancement, the challenges these partnerships face, and the emerging best practices. This review followed Arksey and O’Malley’s framework and the PRISMA-ScR guidelines. We systematically searched peer-reviewed databases, including PubMed, Web of Science, Scopus, and CINAHL, for relevant studies published from inception until December 2024. Data were extracted and analyzed thematically using NVivo 12 to identify key themes related to state-church partnership models, their impact on UHC, implementation challenges, and emerging best practices. The review included eight studies which revealed that FBOs contribute between 30% and 70% of healthcare services in some regions, improving access, affordability, and equity. They play a critical role in maternal and child health, HIV/AIDS prevention, and health workforce training. However, challenges such as funding constraints, service quality variability, and limited policy integration hinder their effectiveness. Emerging best practices include enhanced government collaboration, community engagement, and capacity-building initiatives. In conclusion, State-Church Partnerships are vital in strengthening healthcare systems and achieving UHC in SSA. To maximize their impact, formalized policy frameworks, sustainable financing mechanisms, and quality assurance measures are essential. Strengthening state-FBO collaboration can bridge healthcare gaps and ensure equitable healthcare access.

Key messages

- Faith-based organizations (FBOs) provide 30%–70% of health services in remote and hard-to-reach areas of SSA, significantly improving care for communicable and non-communicable diseases, including maternal and child health and HIV/AIDS.
- Funding instability and variable service quality undermine the effectiveness of state-church partnerships, threatening sustainable progress toward universal health coverage goals.
- Formal policy frameworks, sustainable financing, and FBOs integration into national health systems are essential in maximizing healthcare impact and outcome.
- Investing in capacity building, such as training the workforce to address gaps, improving financial management, and leveraging community ties, enhances service uptake, trust, and quality.

## Introduction

Universal Health Coverage (UHC) remains a critical global health goal, particularly in sub-Saharan Africa (SSA) (Ifeagwu et al. 2021, Arhin et al. 2023), where healthcare systems face persistent challenges such as limited infrastructure, access to quality healthcare, inadequate funding, and workforce shortages (Langat et al. 2025). These challenges contribute to significant gaps in service delivery and coverage, especially in rural and hard-to-reach areas (Strasser et al. 2016, Afriyie et al. 2025). In this context, innovative strategies that leverage existing resources and partnerships emerge as potential solutions to accelerate progress toward UHC (Derose et al. 2011, Bielefeld and Cleveland 2013). One such strategy is the collaboration between the state and religious institutions, often referred to as State-Church Partnerships (SCPs) (Felix 2011, Halmai 2017).

Faith-based organizations (FBOs) have historically played a pivotal role in healthcare delivery in SSA, operating hospitals, clinics, and community health programs that complement government efforts (Grieve and Olivier 2018, Nicol et al. 2022). Their extensive networks and deep-rooted community presence enable them to provide essential, people-centered care, particularly in remote and marginalized regions. Some estimates suggest that FBOs deliver between 30% and 70% of all healthcare services in some SSA countries, highlighting their significant role in national health systems (The United States Agency for International Development 2019).

Recognizing this potential, governments in SSA have increasingly sought to formalize partnerships with religious institutions to strengthen healthcare systems and expand access to care (Green et al. 2002, Boulenger et al. 2012). For instance, Kenya's Ministry of Health has recently emphasized closer collaboration with FBOs, introducing legislative reforms like the Social Health Insurance Act of 2023 to ensure sustainable financing (Kenya 2024). However, these partnerships often face challenges, including a lack of FBO representation in policy meetings and a need for support in financial management and compliance (World Health Organization 2021, Ala et al. 2025). This gap between policy ambition and practical implementation underscores the need for a synthesized evidence base on effective partnership models.

While some studies highlight the contributions of FBOs, there is limited synthesized evidence on how SCPs operate, their impact on UHC goals, and the barriers they face (Grieve and Olivier 2018, The United States Agency for International Development 2019, Nicol et al. 2022). This scoping review, therefore, aims to examine: (i) the existing models of State-Church Partnerships in healthcare delivery within SSA and their impact on UHC advancement, (ii) the challenges these partnerships face, and (iii) the emerging best practices that can inform future policy and practice. The findings are expected to provide a comprehensive overview to support strategic planning and policymaking for sustainable and equitable healthcare systems in SSA.

## Methods

Our study utilized the scoping review following the methodological framework established by Arksey and O'Malley (Arksey and O'Malley 2005). To ensure methodological rigor, we developed a comprehensive protocol for this study (Supplementary Appendix S1). This review report adhered to the PRISMA2020 statement: An updated guideline for Preferred Reporting Systematic Reviews and Meta-Analyses extension for Scoping Reviews (PRISMA-ScR) checklist (Page et al. 2021), which outlines essential items for transparent reporting in scoping reviews. The completed checklist is available in Supplementary Table S1.

### Search strategy

A comprehensive search was conducted in major English peer-reviewed databases, including PubMed, Web of Science, ScienceDirect, Scopus, and Cumulative Index to Nursing and Allied Health Literature, in October 2024 to identify relevant publications from inception until December 2024. For searches in English databases, we used the following terms “State and Church Partnership” OR “FBOs” OR “faith based organization” OR “religious health agency” OR “church healthcare service” OR “faith inspired healthcare” OR “religious health networks” AND “Healthcare delivery” OR “Health services” OR “Health systems” OR “Primary healthcare” OR “Community health” OR “Health infrastructure” AND “Universal Health Coverage” OR “UHC” OR “Health for all” OR “Equitable healthcare” OR “Health equity” AND “Sub-Saharan Africa” OR “Africa” OR “SSA”. The search string was first tested in PubMed and subsequently adapted to other databases. The search strings used across all databases are detailed in Supplementary Tables S2.1–S2.5. Additionally, the reference lists of included publications were screened for relevant articles not otherwise captured.

### Inclusion and exclusion criteria

The review included English publications that provided qualitative and quantitative evidence on the state-church partnerships. We specifically targeted articles detailing (i) partnerships between state entities and church/faith-based organizations addressing innovative healthcare delivery strategies toward UHC; (ii) research conducted in SSA; (iii) study types: empirical studies (qualitative, quantitative, mixed-methods), reviews, policy analyses, and case studies. Articles not directly related to state-church partnerships, general discussions without a specific focus solely on other types of public-private collaborations, opinion pieces, commentaries, editorials, letters, and studies lacking sufficient methodological detail were excluded.

### Study selection

All imported records were retrieved into EndNote X9 software (Clarivate, Philadelphia, USA), and duplicates were removed. The inclusion and exclusion criteria were pilot-tested on 25% of the articles and refined for clarity and applicability. Three reviewers independently screened the titles and abstracts of the English publications and then compared their results. A fourth reviewer performed similar tasks and validated the selections after our pilot test. The research team regularly consulted with experience researchers in review methodology, with much emphasis on empirical research and systematic reviews. To resolve discrepancies, regular meetings were held to reach consensus before proceeding to the next stage. This approach, which involved multiple screeners, was pivotal in reducing biases and errors (Peters et al. 2021).

### Quality appraisal of included publications

The quality of included articles was appraised using the Joanna Briggs Institute (JBI) Critical Appraisal Checklist (Joanna Briggs Institute 2020) to characterize the methodological strengths and limitations of the evidence base, in line with the descriptive analytical purpose of a scoping review. Each criterion on the checklist was evaluated for every study, assigning ratings of “Yes”, “No,” “Unclear,” or “Not applicable.” Articles without any “No” or “Unclear” ratings were considered “Strong” in quality. Those with one to three such ratings were categorized as “Moderately strong,” while those with more than three were deemed “Weak.” However, consistent with scoping review methodology, all studies that met the inclusion criteria were retained for analysis regardless of their quality appraisal rating. The appraisal results are presented descriptively in the results section, and detailed appraisals of each publication are available in Supplementary Tables S3.1–S3.3.

### Data extraction

A purposefully designed Microsoft Excel spreadsheet was used to extract data from included articles, including title, year of publication, first or corresponding author’s country, journal impact factor, article type, and study design, data source, targeted country, targeted issue, and reported outcomes. Two reviewers first tested the data extraction form on three English articles individually and consulted with a third reviewer to resolve discrepancies. Afterwards, the two reviewers extracted all data from the English articles.

### Evidence synthesis and analysis

Thematic analysis was employed to examine the data (Clarke and Braun 2017), using NVivo 12 qualitative data analysis software (QSR International, Burlington, USA) to organize the included articles (NVivo). The analysis followed an inductive approach, allowing themes to emerge from the data. Three reviewers coded the included English articles, met regularly to compare their coding frameworks and discuss discrepancies until a consensus was reached on the final thematic structure. To ensure the trustworthiness and validity of the analytical processes, a debriefing technique was employed (Chereni et al. 2020).

## Results

### Selection of sources of evidence

**Figure 1.**
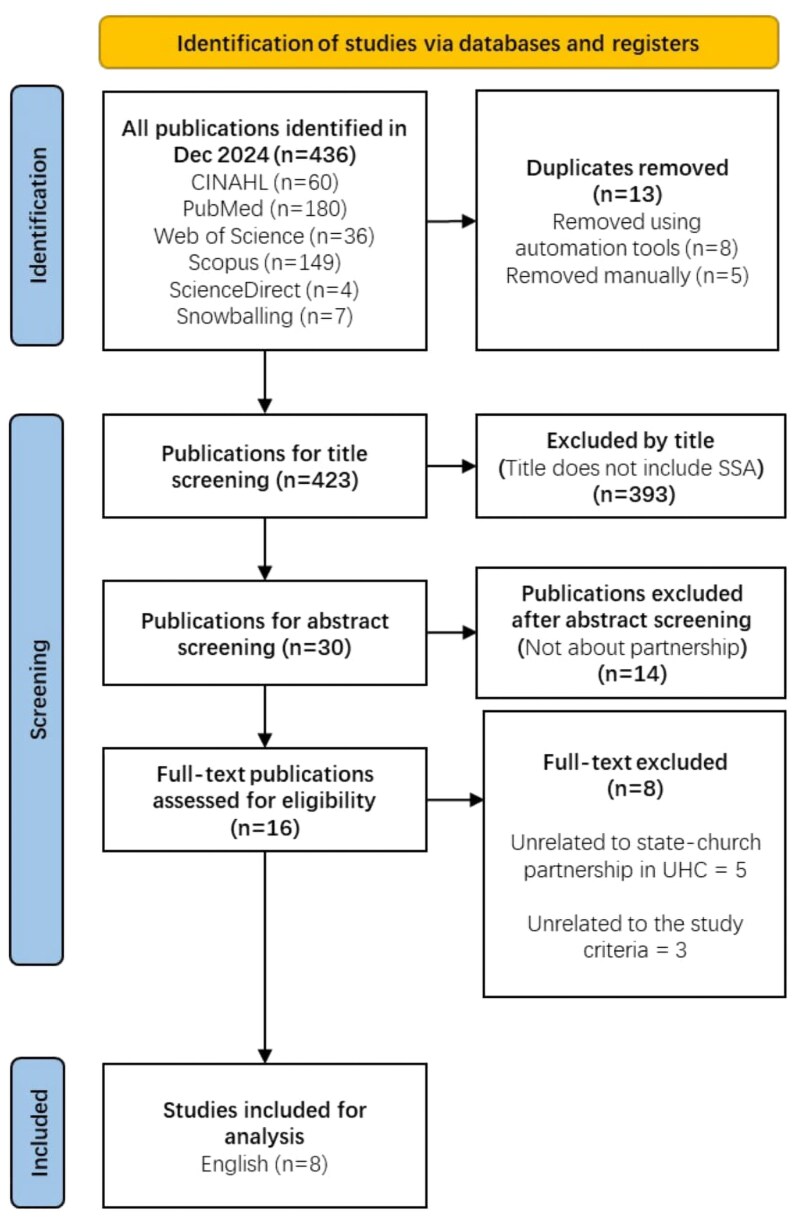
PRISMA-ScR flow diagram of the study selection process.

The study selection process is illustrated in the PRISMA-ScR flow diagram in Fig. 1. Initially, a total of 436 English publications were identified. After duplicates were removed, the numbers decreased to 423. Subsequent title and abstract screening further narrowed the selection to 16 English articles for full-text review. Finally, eight English articles (five unrelated to state-church partnership, and three unrelated to the study) were excluded as they did not meet the inclusion criteria. This resulted in eight English articles for the final inclusion and synthesis.

### Characteristics of literature included

Table 1 outlines the characteristics of the 8 English articles included in our analysis, published between 2011 and 2023. Most of these articles (*n* = 5) explored the role of FBOs across multiple African countries, while two articles focused on individual countries: Tanzania and Ghana. Researchers from high-income countries (HICs) (e.g. UK, Belgium, Switzerland, Sweden), were the first or corresponding authors in five of the eight articles, while three articles were published collaboratively by researchers from SSA, such as South Africa, Uganda, and Cameroon. Three articles were published in journals with impact factors below 1.5, while five articles appeared in journals with an impact factor between 2.5 and 3.5.

**Table 1.** Characteristics of the eight English articles included for evidence synthesis and analysis.

Title	Year of publication	First or corresponding author’s country	Journal (IF in the year of publication)	Article type and study design	Data source	Targeted country	Targeted issue	Reported outcomes
The impact of faith-based organizations on maternal and child health care outcomes in Africa: taking stock of research evidence (Nicol et al. 2022)	2022	Cape Town, South Africa	0.96	Review	Literature review	Africa	Maternal and child health care outcomes	Improved access and delivery of MCH services and the potential of strengthening the health system at large
Contracting between Faith-based health care Organizations and the Public sector in Africa (Boulenger et al. 2012)	2012	Uganda	0.01	Review	Semi-structured interviews (100 interviews), desk review, additional interviews	Anglophone (Tanzania and Uganda) and Francophone (Cameroon and Chad) countries	Contracting between faith-based health organizations and the public health sector for healthcare service delivery	Contracted arrangements for integrating faith-based health facilities into national healthcare systems
Aligning faith-based and national HIV/AIDS prevention responses? Factors influencing the HIV/AIDS prevention policy process and response of faith-based NGOs in Tanzania (Morgan et al. 2014)	2014	UK	3.33	Original research: Qualitative	72 semi-structured interviews, document review and observation	Tanzania	Factors influencing the HIV/AIDS prevention policy process	HIV/AIDS prevention strategies employed by faith-based organisations, including their positions on condom use and the implications for national health response efforts
Faith based organizations: Types and Typologies. A scoping review (2010–2021) (Maes et al. 2023)	2023	Belgium	0.95	Review	Literature review	Global scope	Understanding typologies of faith-based organisations involved in poverty alleviation	Development of a typology framework for classifying FBOs
The role of faith-based organizations in maternal and newborn health care in Africa (Widmer et al. 2011)	2011	Geneva, Switzerland	1.96	Review	Literature review	Ghana, Malawi, Mozambique, Nigeria, Uganda, and Tanzania	Maternal and newborn health care disparities and the role of faith-based organizations in service delivery	FBOs provides health interventions including prenatal care, skilled delivery services, postnatal care, immunization, and health education through community-based programs
The role of faith-based health professions schools in Cameroon’s health system (Herzig van Wees et al. 2021)	2021	Sweden	3.33	Original research: Qualitative study	24 qualitative interviews and 3 focus group discussions involving various stakeholders	Cameroon	The impact of faith-based health professions schools on Cameroon’s health system	Training of health professionals in faith-based institutions
Toward universal health coverage: a mixed-method study mapping the development of the faith-based non-profit sector in the Ghanaian health system (Grieve and Olivier 2018)	2018	Cape Town, South Africa	2.49	Original research: Mixed methods	Geospatial data from the Centre for Remote Sensing and Geographic Information Services (CERSGIS) CHAG (Christian Health Association of Ghana) membership lists WHO Service Availability Mapping report Archival documents, organizational reports, and key informant interview**s**	Ghana	Inequitable geographical distribution of health services and the role of faith-based non-profit providers in achieving UHC	Mapping and analysis of the historical and current spatial distribution of faith-based non-profit health providers in Ghana Collaboration between public sector and FBNPs to strengthen the health system and improve access to healthcare, particularly in underserved rural areas Examination of the role of FBNPs in addressing geographic access and contributing to UHC
The challenges of donor engagement with faith-based organizations in Cameroon’s health sector: a qualitative study (van Wees and Jennings 2021)	2021	Sweden, UK	3.5	Original research: Qualitative Study	46 in-depth interviews with Ministry of Health officials, FBOs, and donors (2015–2017)	Cameroon	Donor-FBO partnerships in health sector	Examined implications of donor engagement on health system strengthening, highlighting policy gaps and unintended consequences (e.g. parallel systems, faith-blind approaches).

CHAG, Christian Health Association of Ghana; FBNP, Faith-based Non-profit Provider; FBO, Faith-based Organization; HBC, home-based care; IF, impact factor; MCH, Maternal and Child Health; SSA, Sub-Saharan Africa; UHC, Universal Health Coverage; VCT, Voluntary Counseling and Testing.

The included articles comprised of four reviews and four original research studies, of which three employed qualitative methods and 1 used a mixed-method approach. While the inclusion of review articles (*n* = 4) is consistent with the aim of a scoping review to map all relevant literature, we note that their findings are interpretations of primary studies. These studies addressed a broad spectrum of health-related issues in African countries, highlighting the multifaceted contributions of FBOs across the continent. Key topics included maternal and child health (MCH) (*n* = 3), HIV/AIDS prevention (*n* = 1), UHC (*n* = 1), health system strengthening (*n* = 3), contracting arrangements (*n* = 1), and FBO typologies (*n* = 1). The interventions undertaken by FBOs, as reported in the articles, included community-based health services, health workforce training, policy influence, and geospatial mapping of health services.

### Overview of thematic findings

The thematic analysis of the eight included articles revealed four key themes that structure the presentation of our findings; (i) The role of partnerships in healthcare delivery: public-private partnerships and faith-based organizations; (ii) The impact on UHC: improved access to healthcare, affordability, equity in service delivery; (iii) Challenges in implementation and sustainability: funding constraints, variability in service quality; and (iv) Emerging best practices: community engagement, training of healthcare workers. Table 2 provides details of the findings within each of these thematic areas.

**Table 2.** Summary of key findings from eight reviewed articles.

Research objective	Emerging theme(s)	No of articles	Reference(s)	Main findings
Role of partnerships in healthcare delivery	Public–private partnerships	4	(Widmer et al. 2011, Boulenger et al. 2012, van Wees and Jennings 2021, Nicol et al. 2022)	FBOs deliver between 30% and 70% of health services in SSA, including MCH services. They play a critical role in healthcare delivery, especially in rural areas, often in partnership with public health systems.
	Faith-based organizations	5	(Widmer et al. 2011, Boulenger et al. 2012, Morgan et al. 2014, van Wees and Jennings 2021, Nicol et al. 2022)	FBOs operate in partnership with government health systems, providing primary, secondary, and tertiary care, especially to poor populations. HIV/AIDS prevention and mitigation by providing services such as voluntary counseling and testing (VCT), home-based care (HBC), and prevention education.
Impact on UHC	Improved access to healthcare	6	(Widmer et al. 2011, Boulenger et al. 2012, Morgan et al. 2014, Grieve and Olivier 2018, van Wees and Jennings 2021, Nicol et al. 2022)	Contribute to reducing maternal and child mortality by providing affordable and accessible healthcare services. Improved access to healthcare, particularly in marginalized rural areas where government services are scarce. Contribute to improving access to HIV/AIDS prevention and care services, particularly in areas where government services are limited.
	Affordability	3	(Widmer et al. 2011, Boulenger et al. 2012, Nicol et al. 2022)	Subsidized services for malaria treatment and immunizations reduced out-of-pocket costs for patients, improving affordability. Provision of affordable healthcare services, often at lower costs compared to public facilities, making healthcare more accessible to poor populations.
	Equity in service delivery	6	(Widmer et al. 2011, Boulenger et al. 2012, Morgan et al. 2014, Grieve and Olivier 2018, van Wees and Jennings 2021, Nicol et al. 2022)	Provision of higher quality care compared to government facilities, leading to increased patient satisfaction and trust. Provision of services to all individuals, regardless of religious affiliation, contributing to equity in service delivery.
Challenges in implementation and sustainability	Funding constraints	7	(Boulenger et al. 2012, Morgan et al. 2014, Grieve and Olivier 2018, Herzig van Wees et al. 2021, van Wees and Jennings 2021, Nicol et al. 2022, Maes et al. 2023)	Significant funding challenges, as public subsidies are often insufficient, delays leading to financial strain and reliance on external donors.
	Variability in service quality	5	(Widmer et al. 2011, Boulenger et al. 2012, Morgan et al. 2014, van Wees and Jennings 2021, Nicol et al. 2022)	Evidence lacking on the cost-effectiveness and long-term sustainability of FBO interventions including the need for capacity building, research collaboration, and funding to support FBO-led healthcare initiatives.
Emerging best practices	Community engagement	5	(Widmer et al. 2011, Boulenger et al. 2012, Morgan et al. 2014, Grieve and Olivier 2018, Nicol et al. 2022)	Engagement in community outreach and health education, which increases trust and utilization of healthcare services.
	Training of healthcare workers	6	(Widmer et al. 2011, Boulenger et al. 2012, Morgan et al. 2014, Herzig van Wees et al. 2021, Nicol et al. 2022, Maes et al. 2023)	Training programs for healthcare workers and community health volunteers to improve clinical skills and quality of care.

FBO, Faith-based Organization; HBC, Home-Based Care; MCH, Maternal and Child Health; SSA, Sub-Saharan Africa; UHC, Universal Health Coverage; VCT, Voluntary Counseling and Testing.

### Role of partnerships in healthcare delivery

#### Public–private partnerships

Formal public–private partnerships (PPPs) were identified as a critical mechanism for integrating FBOs into national health systems, particularly in countries like Tanzania and Uganda. The core of these models involved formal contracting frameworks that enabled governments to subsidize care and share infrastructure with FBO-run facilities (Widmer et al. 2011, Boulenger et al. 2012, van Wees and Jennings 2021, Nicol et al. 2022). For instance, in Uganda, co-funded antenatal care programs through PPPs directly addressed maternal health disparities in rural regions. This collaboration expanded service coverage and improved care quality through shared resources like medical equipment and training modules (Widmer et al. 2011). Similarly, Tanzania leveraged PPPs to combat HIV/AIDS by deploying FBO clinics as hubs for prevention services, including testing and community education. These partnerships ensured that hard-to-reach populations, often excluded from government health networks, gained access to critical interventions, thereby aligning with national UHC goals (Boulenger et al. 2012, van Wees and Jennings 2021).

#### Faith-based organizations

FBOs serve as foundational pillars of healthcare delivery in SSA, bridging critical gaps in access and quality (Widmer et al. 2011, Boulenger et al. 2012, Morgan et al. 2014, van Wees and Jennings 2021, Nicol et al. 2022). Their contribution is substantial; e.g. in Ghana, the Christian Health Association of Ghana (CHAG) delivers 30%–40% of national health services, with a pronounced focus on MCH (Widmer et al. 2011). Through community-based prenatal and postnatal care programs, CHAG clinics improved skilled birth attendance rates and immunization coverage, particularly in remote areas where government facilities were sparse. Beyond direct service provision, FBOs have addressed systemic challenges such as workforce shortages (van Wees and Jennings 2021). In Cameroon, faith-based health professions schools trained nurses and midwives, equipping rural regions with skilled personnel and reducing disparities in healthcare access (Boulenger et al. 2012).

### Impact on UHC

#### Improved access to healthcare

A key finding was the significant enhancement of healthcare access by FBOs, particularly in geographically isolated and underserved regions (Widmer et al. 2011, Boulenger et al. 2012, Morgan et al. 2014, Grieve and Olivier 2018, van Wees and Jennings 2021, Nicol et al. 2022). Geospatial mapping studies revealed that FBO clinics filled critical gaps in rural healthcare infrastructure, mitigating geographic inequities and ensuring remote communities received essential services such as maternal care and disease prevention (Grieve and Olivier 2018, van Wees and Jennings 2021). For example, Tanzania’s FBO-led HIV/AIDS programs increased uptake of voluntary counseling and testing (VCT) services in remote areas (Widmer et al. 2011, Morgan et al. 2014). In Malawi and Nigeria, faith-based antenatal care initiatives increased coverage by 20%–30%, directly contributing to reductions in maternal mortality rates and illustrating the scalability of FBO interventions in resource-limited settings (Widmer et al. 2011).

#### Affordability

FBOs enhance UHC by making healthcare more affordable and reducing financial barriers for low-income and vulnerable groups. This is achieved through subsidized services at faith-based facilities, which lower out-of-pocket expenses for households. In Kenya and Liberia, subsidized services at faith-based facilities such as malaria treatment and childhood immunizations reduced out-of-pocket expenses for households, enabling broader utilization of lifesaving care (Widmer et al. 2011, Boulenger et al. 2012, Nicol et al. 2022). Ghana's CHAG network demonstrated this effectively, as faith-based services often incurred lower out-of-pocket costs compared to public facilities, thereby enhancing affordability for the most vulnerable segments of the population (Widmer et al. 2011).

#### Equity in service delivery

FBOs have consistently prioritized equitable care for marginalized populations, transcending religious and socioeconomic divides (Widmer et al. 2011, Boulenger et al. 2012, Morgan et al. 2014, Grieve and Olivier 2018, van Wees and Jennings 2021, Nicol et al. 2022). A core strength is their ability to tailor services to specific community needs, ensuring access regardless of religious affiliation or geographic isolation. In Cameroon and Tanzania, faith-based clinics tailored services to refugees and rural communities (Boulenger et al. 2012, Morgan et al. 2014). Furthermore, in Ghana, faith-based clinics achieved higher patient satisfaction due to equitable treatment practices, aligning with the core UHC principle of inclusivity (Grieve and Olivier 2018), demonstrating how faith-based actors can bridge disparities and foster cohesive, people-centered health systems (van Wees and Jennings 2021).

### Challenges in implementation and sustainability

#### Funding constraints

Financial instability remains a pervasive barrier to sustaining FBO-led healthcare programs. A common issue is the over-reliance on unpredictable external funding and delays in government subsidies, which disrupt operations and force facilities to ration services. In Uganda, delay in government subsidies for contracted faith-based clinics created significant operational challenges (Boulenger et al. 2012, Morgan et al. 2014, Grieve and Olivier 2018, Herzig van Wees et al. 2021, van Wees and Jennings 2021, Nicol et al. 2022, Maes et al. 2023). Similarly, Ghana’s reliance on external donor funding created vulnerabilities, as shifts in donor priorities jeopardized ongoing MCH programs (Grieve and Olivier 2018). Cameroon’s faith-based health schools exemplified this strain, where inadequate financial support limited their capacity to expand nursing and midwifery training, despite rising demand for skilled rural healthcare workers (Herzig van Wees et al. 2021, van Wees and Jennings 2021).

#### Variability in service quality

Inconsistent adherence to healthcare standards and fragmented coordination with public systems further undermine the effectiveness of FBO initiatives (Widmer et al. 2011, Boulenger et al. 2012, Morgan et al. 2014, van Wees and Jennings 2021, Nicol et al. 2022). This variability can lead to uneven health outcomes and diluted public health impact. In Tanzania, disparities in HIV/AIDS prevention outcomes were linked to uneven compliance with national guidelines across faith-based clinics, where some facilities emphasized abstinence-focused messaging over evidence-based strategies like condom distribution (Widmer et al. 2011). In Malawi, fragmented coordination between FBOs and government maternal health programs led to duplicated efforts and uneven care quality. Rural FBO clinics often lacked standardized protocols for emergency obstetric care, resulting in preventable maternal complications compared to urban public facilities (Boulenger et al. 2012, Morgan et al. 2014, van Wees and Jennings 2021, Nicol et al. 2022).

### Emerging best practices

#### Community engagement

FBOs have leveraged their deep-rooted community ties to design interventions that resonate with cultural values and drive health-seeking behaviors (Widmer et al. 2011, Boulenger et al. 2012, Morgan et al. 2014, Grieve and Olivier 2018, Nicol et al. 2022). Engaging local leaders and tailoring health messages to community norms are key strategies for building trust and improving service uptake. In Ghana, faith-based clinics implemented community health education programs that emphasized the importance of antenatal care, leading to a 35% increase in attendance at rural maternal health facilities (Widmer et al. 2011, Grieve and Olivier 2018). In Tanzania, dialogue platforms between FBOs and policymakers helped bridge gaps in HIV/AIDS prevention strategies (Widmer et al. 2011, Boulenger et al. 2012, Morgan et al. 2014). By integrating faith-based perspectives with national guidelines, these discussions aligned messaging with evidence-based practices, resulting in a 40% rise in clinic visits among high-risk populations (Widmer et al. 2011, Boulenger et al. 2012).

#### Training of healthcare workers

Investing in human capital emerged as a cornerstone of FBO-led health system strengthening. Capacity-building initiatives for healthcare workers and FBO staff are essential for improving quality, ensuring compliance, and promoting sustainability. In Cameroon, faith-based nursing schools revised their curricula to align with national priorities, ensuring graduates were proficient in emergency obstetric care and neonatal resuscitation (Widmer et al. 2011, Boulenger et al. 2012, Morgan et al. 2014, Herzig van Wees et al. 2021, Nicol et al. 2022, Maes et al. 2023). This directly addressed rural workforce shortages and improved the quality of maternal care (Widmer et al. 2011, Morgan et al. 2014). Uganda complemented this approach by training FBO staff in financial management and reporting, enabling clinics to comply with public sector standards and secure government subsidies, thereby enhancing operational efficiency and accountability (Widmer et al. 2011).

## Discussion

The findings of this scoping review emphasize that SCPs are not merely a supplementary feature but a structural component of healthcare systems in SSA, critically advancing progress toward UHC. Our synthesis move beyond cataloging contributions to interpret their significance within the region's complex health landscape. This discussion contextualizes these findings, exploring the paradoxical strength and fragility of SCPs, their tangible impact on UHC pillars, and the systemic reforms required to harness their full potential.

The most salient finding is the indispensable yet precarious role of FBOs. Providing 30%–70% of health services in some regions, they are de facto public health providers, particularly in remote and marginalized areas where state presence is thin. This review confirms that their strength lies in a holistic, community-embedded model of care that integrates health services with social support, fostering unparalleled trust and enabling them to reach populations often excluded from mainstream services, such as men and youth in HIV/AIDS programs (Derose et al. 2011, Grieve and Olivier 2018). However, this critical role creates a systemic dependency. Their heavy reliance on unpredictable external funding and donor priorities (Chowdhury et al. 2020) exposes a fundamental vulnerability in the health system's architecture. This paradox, being both a pillar of service delivery and a node of financial instability, highlights an urgent policy imperative: the transition from ad-hoc collaboration to a formalized, sustainably financed integration within national health systems. The sustainability of UHC gains, especially in maternal and child health, is contingent on resolving this tension.

Regarding their impact on UHC, the value of SCPs is most evident in their advancement of the core tenets of access, affordability, and equity. By extending services to geographically isolated communities, FBOs directly mitigate geographic inequities. More importantly, their frequent provision of subsidized care reduces out-of-pocket expenditures for low-income households, addressing a primary financial barrier to access (Ifeagwu et al. 2021). This aligns perfectly with the equity goals of UHC. However, our synthesis reveals a critical nuance: this positive impact is not universal. Variable service quality and fragmented coordination with public systems can inadvertently perpetuate inequities. Disparities in adherence to national clinical guidelines, such as those observed in HIV prevention strategies in Tanzania or emergency obstetric care in Malawi, create a postcode lottery of care quality (Morgan et al. 2014, Nicol et al. 2022). This suggests that mere presence is insufficient; impact is maximized only when FBO service delivery is aligned with and accountable to national quality standards. Therefore, the challenge is not just to fund FBOs but to strategically integrate them to ensure consistency and equity in the quality of care across all sectors.

The challenges identified chronic funding instability and variable quality, which are not merely operational hurdles for FBOs but are symptoms of a broader lack of systemic integration. The solution lies in adopting and scaling emerging best practices through coherent policy. For instance, the success of community engagement models in Ghana and Tanzania, which significantly increased service uptake, demonstrates the power of leveraging FBOs’ social capital (Boulenger et al. 2012, Grieve and Olivier 2018). Similarly, capacity-building initiatives in Cameroon and Uganda that trained health workers and administrators in clinical and financial skills show how investing in FBOs’ human capital strengthens the entire health system’s workforce and governance (Widmer et al. 2011, Herzig van Wees et al. 2021). These best practices must be systematized. They point toward the need for formal contracting frameworks that explicitly bundle sustainable financing with obligations for quality assurance, reporting, and professional development (Boulenger et al. 2012). This moves the relationship from a grantor-recipient dynamic to an accountable partnership with shared objectives.

### Policy implications

The findings underscore that maximizing the contribution of SCPs to UHC requires a deliberate shift from informal collaboration to formalized integration. To achieve this, SSA governments must establish structured partnership frameworks that combine sustainable financing with clear quality assurance and reporting obligations, while ensuring FBO representation in policy dialogues. FBO healthcare providers, in turn, must proactively engage in these frameworks, strengthen their internal capacity for financial management and compliance, and ensure service delivery adheres to national clinical guidelines. International donors and agencies can catalyze this process by aligning their funding with national health strategies and supporting long-term, flexible financing that strengthens the overall system. Crucially, this integration must be pursued with a clear-eyed view of potential value conflicts in areas such as reproductive health. Effective policy must therefore mandate that publicly-funded services adhere to national guidelines and guarantee equitable access for all, while constructing mechanisms that respect the religious identity of faith-based partners. Fostering these accountable, strategically-aligned partnerships is essential to connect the extensive reach of FBOs while safeguarding the equity and inclusivity goals at the heart of Universal Health Coverage.

### Strengths and limitations of this review

Our scoping review was strengthened by adhering to established methodological frameworks, such as the Arksey and O’Malley guidelines and the PRISMA-ScR checklist. This ensured a systematic and reliable synthesis of the information. We included only English sources to minimize selection bias and capture a diverse range of perspectives. Additionally, the use of the JBI Checklist enhanced the clarity and credibility of our findings. The reliance on peer-reviewed and review articles may result in the introduction of publication and interpretation bias, potentially leading to an overrepresentation of studies that present favorable outcomes (Song et al. 2013, Chen et al. 2020). Furthermore, excluding studies published after December 2024 may limit the relevance of our findings in light of recent developments. Crucially, the evidence synthesis and conclusions of this review are based on a limited pool of only eight studies. While these studies provide valuable insights, this small number necessarily limits the generalizability of our findings and emphasizes the emerging state of rigorous, published research on State-Church Partnerships in Sub-Saharan Africa. This limitation highlights a significant gap in the literature and points to a pressing need for more primary research to build a more robust evidence base on the models, impacts, and sustainability of SCPs.

## Supplementary Material

czaf082_Supplementary_Data

## Data Availability

The case information sources are listed in Additional File 1: Appendix, and all data and materials used can be accessed from the corresponding authors upon reasonable request.

## Conclusion

This review affirms that SCPs are a powerful, innovative strategy for healthcare delivery in SSA. Their community trust, extensive reach, and holistic care models are assets that no health system can afford to overlook. Yet, their current potential is constrained by systemic fragilities. The path forward requires a deliberate shift from leveraging FBOs as stopgap providers to empowering them as fully integrated, accountable, and sustainably resourced partners within a unified health system. This strategic evolution is essential to translate their substantial contributions into lasting, equitable, and quality health coverage for all.

## Reflexivity statement

The authors represent diverse academic, professional, and geographical backgrounds, which informed the perspective of this research. The lead author (J.A.A.) is a health professional affiliated with both a faith-based organization (FBO) facility and Kwame Nkrumah University of Science and Technology (KNUST), Ghana. Co-authors P.A.B. and K.M.A. specialize in health management and health economics, respectively. Additional contributors (E.K.A. and M.H.K.) are health professionals from a teaching hospital and a Ghana Health Service facility. This combination of clinical, managerial, and economic expertise ensured a multidisciplinary and balanced approach to the study.

## Abbreviations

CHAG: Christian Health Association of Ghana
FBO: Faith-Based Organization
HBC: Home-Based Care
HICs: High-Income Countries
HIV/AIDS: Human Immunodeficiency Virus/Acquired Immune Deficiency Syndrome
JBI: Joanna Briggs Institute
MCH: Maternal and Child Health
PRISMA-ScR: Preferred Reporting Items for Systematic Reviews and Meta-Analyses Extension for Scoping Reviews
SCP: State-Church Partnership
SSA: Sub-Saharan Africa
UHC: Universal Health Coverage
VCT: Voluntary Counseling and Testing
WHO: World Health Organization
