# Supplementary material for: State-church partnerships as an innovative strategy in healthcare delivery for universal health coverage in sub-Saharan Africa: a scoping review
Source: Health Policy Plan. 2025 Oct 29;41(2):275–85. doi: 10.1093/heapol/czaf082 (PMC12906754; doi:10.1093/heapol/czaf082)
Supplement: czaf082_Supplementary_Data [file czaf082_supplementary_data.docx]

**Supplementary Material**

**Appendix**

**Table of Contents**

[Protocol Title: State-Church Partnerships as an Innovative Strategy in Healthcare Delivery for Universal Health Coverage in sub-Saharan Africa: A Scoping Review Protocol 1](#_Toc208482722)

[Background 1](#_Toc208482723)

[Central question 1](#_Toc208482724)

[Review objectives 1](#_Toc208482725)

[Methods 2](#_Toc208482726)

[Overview & Justification 2](#_Toc208482727)

[Inclusion and exclusion criteria 2](#_Toc208482728)

[Research details: 2](#_Toc208482729)

[Search strategy 3](#_Toc208482730)

[Data extraction 3](#_Toc208482731)

[Supplementary Table 1: Preferred Reporting Items for Systematic reviews and Meta-Analyses extension for Scoping Reviews (PRISMA-ScR) Checklist 3](#_Toc208482732)

[Supplementary Table 2: Search Strategy and Results 6](#_Toc208482733)

[Supplementary Table 3: JBI Critical Appraisal Checklist 8](#_Toc208482734)

# Protocol Title: State-Church Partnerships as an Innovative Strategy in Healthcare Delivery for Universal Health Coverage in sub-Saharan Africa: A Scoping Review Protocol

## Background

Universal Health Coverage (UHC) remains a critical goal in Sub-Saharan Africa (SSA), where health systems face persistent challenges such as limited infrastructure, funding, and healthcare workforce shortages. Despite efforts by governments and international bodies, gaps in healthcare access—especially in rural and underserved areas—persist. In this context, State-Church Partnerships (SCPs) have emerged as a promising strategy to bolster healthcare delivery. Faith-Based Organizations (FBOs), historically vital in SSA’s health systems, provide 20–40% of healthcare services in several countries, reaching marginalized populations with people-centered and holistic care. Governments increasingly recognize the role of FBOs in achieving UHC, formalizing partnerships through initiatives such as Kenya's Social Health Insurance Act (2023). However, challenges remain, including limited representation of FBOs in policymaking and the need for capacity building in financial and administrative areas. Despite their contributions, the scope, effectiveness, and sustainability of SCPs remain underexplored in literature. This review seeks to examine models of SCPs, their impact on UHC, associated challenges, and best practices to inform policy and identify research gaps.

## Central question

To describe and systematically consolidate existing literature on state-church partnerships in the implementation of healthcare delivery in LMICs in mitigating healthcare delivery, identify the primary facilitators and barriers to their activities, and the knowledge gaps that may usefully be addressed by future research.

###

### Review objectives

To better explain state-church partnership in addressing research questions, the following specific objectives were framed on the following:

1. The existing models of State-Church Partnerships in healthcare delivery within Sub-Saharan Africa, and how they impact the advancement of UHC;
2. Challenges these partnerships face in implementing and sustaining healthcare initiatives, and what best practices have emerged to inform future healthcare policies and practices.

## Methods

### Overview & Justification

We plan to use the scoping review methodology, as described in the work of Arksey and O'Malley, to analyse both qualitative and quantitative evidence on the state-church partnership in addressing UHC in LMICs. Our study protocol will follow the Preferred Reporting Items for Systematic Reviews and Meta-Analyses (PRISMA-ScR) extension for the scoping reviews checklist. Adopting this type of review is useful for “identifying the extent and range of research available on a particular subject, such as state-church partnerships in UHC, offering a clear picture of the amount of existing literature and the focus of the studies. Scoping reviews are particularly valuable for exploring new evidence in areas where it is not yet clear what specific questions should be addressed by a more detailed systematic review.

### Inclusion and exclusion criteria

We will include studies published in English that provide qualitative and quantitative evidence on the state-church partnership in UHC. We will specifically target publications detailing 1) partnerships between state entities and church/faith-based organizations addressing innovative healthcare delivery strategies toward UHC; 2) research conducted in SSA; 3) study types: empirical studies (qualitative, quantitative, mixed-methods), reviews, policy analyses, and case studies. This will be developed based on the selection criteria by the Population, Concept, Context (PCC) framework. We will exclude studies not directly related to state-church partnership, general discussions about state-church partnership without a specific focus, conference proceedings, commentaries, abstracts, book reviews, and papers for which full texts were not accessible.

### Research details:

a) Language: English;

b) Published Time: Inception-November, 2024;

c) Qualitative and quantitative evidence on the state-church partnership addressing innovative healthcare delivery strategies toward UHC;

d) Research conducted in SSA

e) Challenges these partnerships encounter in healthcare delivery.

### Search strategy

- *Database*

English peer-reviewed databases, including PubMed, Web of Science, ScienceDirect, Scopus, and Cumulative Index to Nursing and Allied Health Literature.

*-Search keyword*

Free-text terms "State and Church Partnership" OR "FBOs" OR "faith based organization" OR "religious health agency" OR "church healthcare service" OR "faith inspired healthcare" OR "religious health networks" AND "Healthcare delivery" OR "Health services" OR "Health systems" OR "Primary healthcare" OR "Community health" OR "Health infrastructure" AND "Universal Health Coverage" OR "UHC" OR "Health for all" OR "Equitable healthcare" OR "Health equity" AND "Sub-Saharan Africa" OR "Africa" OR "SSA". We first tested the search string in PubMed and, after validating, will be adapted to other databases.

### Data extraction

We will follow the PRISMA guidelines to conduct the study. Data from the included studies will be extracted by one reviewer and then checked by a second reviewer. We will extract essential information from the studies, and the data extraction table will be provided at a later stage.

## Supplementary Table 1: Preferred Reporting Items for Systematic reviews and Meta-Analyses extension for Scoping Reviews (PRISMA-ScR) Checklist

| **SECTION** | **ITEM** | **PRISMA-ScR CHECKLIST ITEM** | **REPORTED ON**  **PAGE #** |
| --- | --- | --- | --- |
| **TITLE** | | | |
| Title | 1 | Identify the report as a scoping review. | Title page (Page 1) |
|  | | | |
| Structured summary | 2 | Provide a structured summary that includes (as applicable): background, objectives, eligibility criteria, sources of evidence, charting methods, results, and conclusions that relate to the review questions and objectives. | Abstract  (Page 2) |
|  | | | |
| Rationale | 3 | Describe the rationale for the review in the context of what is already known. Explain why the review questions/objectives lend themselves to a scoping review approach. | Page 3-5 |
| Objectives | 4 | Provide an explicit statement of the questions and objectives being addressed with reference to their key elements (e.g., population or participants, concepts, and context) or other relevant key elements used to conceptualize the review questions and/or objectives. | Page 5 |
| **METHODS** | | | |
| Protocol and registration | 5 | Indicate whether a review protocol exists; state if and where it can be accessed (e.g., a Web address); and if available, provide registration information, including the registration number. | Page 6 |
| Eligibility criteria | 6 | Specify characteristics of the sources of evidence used as eligibility criteria (e.g., years considered, language, and publication status), and provide a rationale. | Page 6-7 |
| Information sources* | 7 | Describe all information sources in the search (e.g., databases with dates of coverage and contact with authors to identify additional sources), as well as the date the most recent search was executed. | Page 6-7 |
| Search | 8 | Present the full electronic search strategy for at least 1 database, including any limits used, such that it could be repeated. | Page 6-7 Supplementary File- Appendix 3 |
| Selection of sources of evidence† | 9 | State the process for selecting sources of evidence (i.e., screening and eligibility) included in the scoping review. | Page 7 |
| Data charting process‡ | 10 | Describe the methods of charting data from the included sources of evidence (e.g., calibrated forms or forms that have been tested by the team before their use, and whether data charting was done independently or in duplicate) and any processes for obtaining and confirming data from investigators. | Page 8 |
| Data items | 11 | List and define all variables for which data were sought and any assumptions and simplifications made. | Page 8 |
| Critical appraisal of individual sources of evidence§ | 12 | If done, provide a rationale for conducting a critical appraisal of included sources of evidence; describe the methods used and how this information was used in any data synthesis (if appropriate). | Page 7 |
| Synthesis of results | 13 | Describe the methods of handling and summarizing the data that were charted. | Page 8 |
| **RESULTS** | | | |
| Selection of sources of evidence† | 14 | Give number of sources of evidence screened, assessed for eligibility, and included in the review, with reasons for exclusions at each stage, ideally using a flow diagram. | Page 8-13 |
| Characteristics of sources of evidence | 15 | For each source of evidence, present characteristics for which data were charted and provide the citations. | Page 9 |
| Critical appraisal within sources of evidence | 16 | If done, present data on critical appraisal of included sources of evidence (see item 12). | Supplementary File 1-Appendix 4 |
| Results of individual sources of evidence | 17 | For each included source of evidence, present the relevant data that were charted that relate to the review questions and objectives. | Page -9-13 |
| Synthesis of results | 18 | Summarize and/or present the charting results as they relate to the review questions and objectives. | Page 9-13 |
| **DISCUSSION** | | | |
| Summary of evidence | 19 | Summarize the main results (including an overview of concepts, themes, and types of evidence available), link to the review questions and objectives, and consider the relevance to key groups. | Page 14-17 |
| Limitations | 20 | Discuss the limitations of the scoping review process. | Page 17-18 |
| Conclusions | 21 | Provide a general interpretation of the results with respect to the review questions and objectives, as well as potential implications and/or next steps. | Page 18 |
| **FUNDING** | | | |
| Funding | 22 | Describe sources of funding for the included sources of evidence, as well as sources of funding for the scoping review. Describe the role of the funders of the scoping review. | Title page |

JBI = Joanna Briggs Institute; PRISMA-ScR = Preferred Reporting Items for Systematic reviews and Meta-Analyses extension for Scoping Reviews.

* Where *sources of evidence* (see second footnote) are compiled from, such as bibliographic databases, social media

platforms, and Web sites.

† A more inclusive/heterogeneous term used to account for the different types of evidence or data sources (e.g., quantitative and/or qualitative research, expert opinion, and policy documents) that may be eligible in a scoping review as opposed to only studies. This is not confused with *information sources* (see first footnote).

‡ The frameworks by Arksey and O’Malley (6) and Levac and colleagues (7) and the JBI guidance (4, 5) refer to the process of data extraction in a scoping review as data charting*.*

§ The process of systematically examining research evidence to assess its validity, results, and relevance before using it to inform a decision. This term is used for items 12 and 19 instead of "risk of bias" (which is more applicable to systematic reviews of interventions) to include and acknowledge the various sources of evidence that may be used in a scoping review (e.g., quantitative and/or qualitative research, expert opinion, and policy document).

*From:* Tricco AC, Lillie E, Zarin W, O'Brien KK, Colquhoun H, Levac D, et al. PRISMA Extension for Scoping Reviews (PRISMAScR): Checklist and Explanation. Ann Intern Med.

## Supplemtary Table 2: Search Strategy and Results

**Supplementary Table 2.1: PubMed search strategy** (PubMed, 30/12/2024 03:33:22) **(n=180)**

| **Search number #** | **Search Query** | **Results** |
| --- | --- | --- |
| #1 | "State and Church Partnership" OR "FBOs" OR "faith based organization" OR "religious health agency" OR "church healthcare service" OR "faith inspired healthcare" OR "religious health networks" | 4,283 |
| #2 | "Healthcare delivery" OR "Health services" OR "Health systems" OR "Primary healthcare" OR "Community health" OR "Health infrastructure" | 776,900 |
| #3 | "Universal Health Coverage" OR "UHC" OR "Health for all" OR "Equitable healthcare" OR "Health equity" AND "Sub-Saharan Africa" OR "Africa" OR "SSA" | 325,650 |
|  | **(#1) AND (#2) AND (#3)** | **180** |

**Supplementary Table 2.2: Web of Science search strategy** (Wednesday, December 11/12/ 2024 08:40:00) (**n=11)**

| **Search Category #** | **Search Query** | **Results** |
| --- | --- | --- |
| #1 | TS= ("State and Church Partnership" OR "FBOs" OR "faith based organization" OR "religious health agency" OR "church healthcare service" OR "faith inspired healthcare" OR "religious health networks") | 210 |
| #2 | TS= ("Healthcare delivery" OR "Health services" OR "Health systems" OR "Primary healthcare" OR "Community health" OR "Health infrastructure") | 982,594 |
| #3 | TS= ("Universal Health Coverage" OR "UHC" OR "Health for all" OR "Equitable healthcare" OR "Health equity" AND "Sub-Saharan Africa" OR "Africa" OR "SSA") | 744,142 |
|  | **(#1) AND (#2) AND (#3)** | **11** |

**Supplementary Table 2.3: Scopus search strategy** (Wednesday, December 11/12/ 2024 08:37:00) **(n=149)**

| **Item** | **Search Query** | **Results** |
| --- | --- | --- |
| 1 | ALL "State and Church Partnership" OR "FBOs" OR "faith based organization" OR "religious health agency" OR "church healthcare service" OR "faith inspired healthcare" OR "religious health networks" | 6,609 |
| 2 | ALL "Healthcare delivery" OR "Health services" OR "Health systems" OR "Primary healthcare" OR "Community health" OR "Health infrastructure" | 2,783,523 |
| 3 | ALL Universal Health Coverage" OR "UHC" OR "Health for all" OR "Equitable healthcare" OR "Health equity" AND "Sub-Saharan Africa" OR "Africa" OR "SSA" | 32,061 |
|  | **1 AND 2 AND 3** | **149** |

**Supplementary Table 2.4: Cumulative Index to Nursing and Allied Health Literature search strategy** (Wednesday, February 11/12/ 2024 08:30:00 **(n= 11)**

| **Search ID #** | **Search Query** | **Actions** |
| --- | --- | --- |
| S1 | TX ("State and Church Partnership" OR "FBOs" OR "faith based organization" OR "religious health agency" OR "church healthcare service" OR "faith inspired healthcare" OR "religious health networks") | (352) |
| S2 | TX ("Healthcare delivery" OR "Health services" OR "Health systems" OR "Primary healthcare" OR "Community health" OR "Health infrastructure") | (846,476) |
| S3 | TX ("Universal Health Coverage" OR "UHC" OR "Health for all" OR "Equitable healthcare" OR "Health equity" AND "Sub-Saharan Africa" OR "Africa" OR "SSA") | (51,554) |
|  | **S1 AND S2 AND S3** | **(11)** |

**Supplementary Table 2.5: ScienceDirect search strategy** (Thursday, February 12/12/ 2024 08:55:00 (n= 4)

| **Search ID #** | **Query** | **Actions** |
| --- | --- | --- |
| ALL | "State and Church Partnership" OR "FBOs" OR "faith based organization" OR "religious health agency" OR "church healthcare service" OR "faith inspired healthcare" OR "religious health networks" AND "Healthcare delivery" OR "Health services" OR "Health systems" OR "Primary healthcare" OR "Community health" OR "Health infrastructure" AND "Universal Health Coverage" OR "UHC" OR "Health for all" OR "Equitable healthcare" OR "Health equity" AND "Sub-Saharan Africa" OR "Africa" OR "SSA" | **4** |

## Supplementary Table 3: JBI Critical Appraisal Checklist

**Supplementary Table 3.1:** JBI Critical Appraisal Checklist for textual evidence: Narrative in 4 studies on state-church partnership in SSA

| Studies | Is the generator of the narrative a credible or appropriate source? | Is the relationship between the text and its context explained? (where, when, who with, how) | Does the narrative present the events using a logical sequence so the reader or listener can understand how it unfolds? | Do you, as reader or listener of the narrative, arrive at similar conclusions to those drawn by the narrator? | Do the conclusions flow from the narrative account? | Do you consider this account to be a narrative? |
| --- | --- | --- | --- | --- | --- | --- |
| Nicol et al. | Yes | Yes | Yes | Yes | Yes | Yes |
| Boulenger et al. | Yes | Yes | Yes | Yes | Yes | Yes |
| Maes et al. | Yes | Yes | Yes | Yes | Yes | Yes |
| Widmer et al. | Yes | Yes | Yes | Yes | Yes | Yes |

**Supplementary Table 3.2:** JBI Critical Appraisal Checklist for qualitative research in 3 studies on state-church partnership in SSA

| Studies | Is there congruity between the stated philosophical  perspective and the research methodology? | Is there congruity between the research methodology and the research question or objectives? | Is there congruity between the research methodology and the methods used to collect data? | Is there congruity between the research methodology and the representation and analysis of data? | Is there congruity between the research methodology and the interpretation of results? | Are participants, and their voices, adequately represented? | Is the research ethical according to current criteria or, for recent studies, and is there evidence of ethical approval by an appropriate body? | Do the conclusions drawn in the research report flow from the analysis, or interpretation, of the data? |
| --- | --- | --- | --- | --- | --- | --- | --- | --- |
| Morgan et al. | Yes | Yes | Yes | Yes | Yes | Yes | Yes | Yes |
| Herzig et al. | Yes | Yes | Yes | Yes | Yes | Yes | Yes | Yes |
| Herzig van Wees & Jennings | Yes | Yes | Yes | Yes | Yes | Yes | Yes | Yes |

**Supplementary Table 3.3:** JBI Critical Appraisal Checklist for analytical cross-sectional studies in 1 study on state-church partnership in SSA

| Studies | Were the criteria for inclusion in the sample clearly defined? | Were the study subjects and the setting described in detail? | Was the exposure measured in a valid and reliable way? | Were objective, standard criteria used for measurement of the condition? | Were confounding factors identified? | Were strategies to deal with confounding factors stated? | Were the outcomes measured in a valid and reliable way? | Was appropriate statistical analysis used? |
| --- | --- | --- | --- | --- | --- | --- | --- | --- |
|  | Yes | Yes | Unclear | Yes | Yes | Not applicable | Yes | Yes |
